# SUMO-Modification of the La Protein Facilitates Binding to mRNA In Vitro and in Cells

**DOI:** 10.1371/journal.pone.0156365

**Published:** 2016-05-25

**Authors:** Venkatesh Kota, Gunhild Sommer, Chantal Durette, Pierre Thibault, Erna A. van Niekerk, Jeffery L. Twiss, Tilman Heise

**Affiliations:** 1 Medical University of South Carolina, Department of Biochemistry & Molecular Biology, Charleston, South Carolina, United States of America; 2 Institute of Research in Immunology and Cancer University de Montreal, Station Centre-ville, Montreal, Canada; 3 Department of Neurosciences-0626, University of California, San Diego, La Jolla, California, United States of America; 4 Department of Biological Sciences, University of South Carolina, Columbia, South Carolina, United States of America; German Cancer Research Center, GERMANY

## Abstract

The RNA-binding protein La is involved in several aspects of RNA metabolism including the translational regulation of mRNAs and processing of pre-tRNAs. Besides its well-described phosphorylation by Casein kinase 2, the La protein is also posttranslationally modified by the Small Ubiquitin-like MOdifier (SUMO), but the functional outcome of this modification has not been defined. The objective of this study was to test whether sumoylation changes the RNA-binding activity of La. Therefore, we established an *in vitro* sumoylation assay for recombinant human La and analyzed its RNA-binding activity by electrophoretic mobility shift assays. We identified two novel SUMO-acceptor sites within the La protein located between the RNA recognition motif 1 and 2 and we demonstrate for the first time that sumoylation facilitates the RNA-binding of La to small RNA oligonucleotides representing the oligopyrimidine tract (TOP) elements from the 5’ untranslated regions (UTR) of mRNAs encoding ribosomal protein L22 and L37 and to a longer RNA element from the 5’ UTR of cyclin D1 (CCND1) mRNA *in vitro*. Furthermore, we show by RNA immunoprecipitation experiments that a La mutant deficient in sumoylation has impaired RNA-binding activity in cells. These data suggest that modulating the RNA-binding activity of La by sumoylation has important consequences on its functionality.

## Introduction

The ubiquitously expressed mammalian La protein (or La-related protein 3, LARP3) is an essential RNA-binding protein (RBP) that plays an important role in cellular and viral RNA metabolism (for review [1–3]). La is predominantly located in the nucleus and nucleolus, but it can also shuttle between the nucleus and the cytoplasm [4–6]. Cytoplasmic La is involved in the translation of a variety of viral [7–9] and cellular mRNAs [10–15]. Additionally, La is also implicated in the translation of mRNAs containing a 5’-terminal oligopyrimidine tract (TOP) encoding ribosomal proteins and translation elongation factors [16, 17]. Binding of La to TOP mRNAs has been connected with promotion [18, 19] and attenuation [20, 21] of TOP mRNA translation. Several RNA immunoprecipitation (RIP) studies [11, 15, 21, 22] suggests that the La protein binds to mRNAs, raising the question, whether the RNA-binding activity of La is regulated by posttranslational modifications. Previously, it has been shown that phosphorylation of La by Casein kinase 2 (CK2) impairs its association with the TOP mRNA L37 [21]. We have recently shown that La is sumoylated [23] and undertook this study to test whether sumoylation modulates its RNA-binding activity.

Small Ubiquitin-like MOdifiers (SUMO) are small proteins (12kDa) that covalently and reversibly modify cellular proteins. The sumoylation of proteins plays an important regulatory role in many biological processes including transcription, RNA processing, cell cycle progression, and stress response [24–28]. In vertebrates, four highly conserved SUMO paralogs are expressed, SUMO-1, SUMO-2, SUMO-3, and SUMO-4. The SUMO-2 and -3 share 95% amino acid sequence homology and differ only in three N-terminal residues in their conjugated forms, whereas SUMO-1 has only 50% sequence identity to the SUMO-2/-3 subfamily [26, 29]. Often only a small fraction of the protein of interest is sumoylated at a given time in cells as well as in vitro [30–33]. Recently, murine and human La have been shown to undergo sumoylation by SUMO-1 and SUMO-2 and it has been shown that sumoylation of La is required for its retrograde transport in axons of rodent sensory neurons [23]. Furthermore, SUMO-2 was conjugated to La in cells after exposure to heat shock [34], and proteomics studies identified the following SUMO-acceptor sites in La: lysines 35, 86, 400 in HeLa cells [35, 36], and lysines 76, 86, 105 and 116 in HEK293 cells [37]. It is not known if these post-translational modifications impact La’s function(s), as any connection between sumoylation of La and its RNA-binding activity still remains unknown. To determine whether sumoylation of La alters its RNA-binding activity, we developed an *in vitro* sumoylation assay. We identified two novel SUMO-acceptors sites on La and show that sumoylation of La facilitates its binding to RNA elements derived from TOP and CCND1 mRNAs. Furthermore, we show by RNA immunoprecipitation experiments that a La mutant deficient in sumoylation has impaired RNA-binding activity. Our findings suggest that sumoylation of La alters its RNA-binding activity, which could influence a broad range of RNA processing steps where La’s function has been implicated.

## Materials and Methods

### Plasmids

The cloning of the plasmid pet28b La has been described [22, 38]. We are thankful to Ron Hay for sharing plasmids with us: pet15 Ubc9 [39], pcDNA SAE1 [40], pcDNA SAE2 [40]. Further, we are indebted to Cheng-Ming Chiang for sharing plasmids: pet11d SUMO-1-GG, pet11d SUMO-2-GG, pet11d SUMO-3-GG, pet11d SUMO-1-GA, pet11d SUMO-2-GA and pet11d SUMO-3-GA [41]. The minimal RNA-binding competent La protein RRM1+2 was created by amplifying nucleotides 340 to 1005 (amino acid 114 to 326) with primers 69S sense primer (spanning nts 314 to 335, start codon is in bold, *EcoR* I restriction site underlined) 5’-GTCCCGAATTCTACCATGAAAAATGATGTAAAAAACAGA-3’ and 70AS antisense primer (spanning nts 984 to 1001, stop codon in bold *Hind* III restriction site underlined) 5’-CGCCCAAAGCTTCTAACGACCTTTTGACTTCCATTT-3’ by using Platinum *Taq* DNA Polymerase High Fidelity [42]. The PCR products were purified using the QIAquick PCR-purification and QIAquick Gel Extraction Kit from Qiagen, analyzed for correct size and purification success on a 1.5% agarose gel, and ligated into the pet28b(+) vector. Point mutations K200R and K200/208R were introduced by PCR-based oligo-directed mutagenesis into La-RRM1+2. Gfp-LaK41/200R was generated by oligo-directed mutagenesis on plasmid gfpLa-K41R [23]. Finally, correct mutagenesis was confirmed by DNA sequencing.

### Recombinant protein expression and purification

*E*. *coli* BL21 cells were transformed with expression vectors encoding His-tagged proteins: His_6_-hLa, His_6_-Ubc9, His_6_-SUMO1-GG, His_6_-SUMO-1GA, His_6_-SUMO2-GG, His_6_-SUMO2-GA, His_6_-SUMO3-GG and His_6_-SUMO3-GA; or GST-tagged protein: GST-SAE1 and GST-SAE2. Pre-cultures were grown overnight in 5 ml lysogeny broth (LB Broth) Miller (EMD) in presence of 100 μg/ml Ampicillin or 30 μg/ml Kanamycin at 37°C. These ‘pre-cultures’ were used to inoculate 2 x 400 ml LB Broth Miller in the presence of 100 μg/ml Ampicillin or 30 μg/ml Kanamycin which were grown at 37°C until the culture reached OD_605nm_ = 0.4. IPTG (OmniPur) was added to a final concentration of 1 mM. The temperature was reduced to 20°C after IPTG induction, cells were grown overnight and pelleted by centrifugation at 4,000 x g. For preparation of His-tagged proteins cells were lysed in 2x 50 ml 50 mM NaPO_4_, 300 mM NaCl, 20 mM Imidazole, 1 mg/ml lysozyme (EMD) and 1% complete protease inhibitor (Roche). For preparation of GST-tagged fusion proteins, cells were lysed in 2x 50 ml 1x Phosphate buffered saline (PBS, 4.3 mM Sodium Phosphate, Dibasic, 137 mM Sodium Chloride, 2.7 mM Potassium Chloride, 1.4 mM Potassium Phosphate, Monobasic) pH 7.4, 1 mg/ml lysozyme, and 1% complete protease inhibitor for 3 h at 4°C, sonicated every 30 min and stored at -80°C. For purification, lysates were spun at 12,000 x g at 4°C for 1 hour, the supernatant was loaded onto a Ni-NTA HiTrap column (5ml, GE Healthcare) equilibrated with loading buffer (20 mM Tris-HCl, 0.25 M NaCl, (pH 8.0)) or GST FF column (5 ml, GE Healthcare) equilibrated with PBS pH 7.4 (His-tagged proteins, GE Healthcare). His-tagged proteins were eluted with 20 mM Tris-HCl, 0.25 M NaCl pH 8.0 at a flowrate of 1.0 ml/min. The elution of GST-fusion proteins was achieved by using 20 mM Tris, 0.1 M NaCl and 10 mM R-glutathione (pH 8.3) with a flowrate of 2.0 ml/min. The proteins were dialyzed against 1000 ml NET-N(S) buffer (50 mM Tris-HCl, 100 mM NaCl, 0.1% Nonidet P-40, 10% glycerol, pH 8.0, 0.5 mM DTT, 1 x protease inhibitor mix) overnight at 4°C. Protein concentrations were determined by UV absorption at 280 nm. To remove bacterial RNA associated with La, His_6_-La was first dialyzed against 50 mM Tris-HCl (pH 7.4), 75 mM sodium chloride, 5 mM calcium chloride and 1x complete protease inhibitor mix (Roche). Subsequently, 500 U Microccocal nuclease (Biolabs) per 1 ml protein solution was added, incubate for 2 h at 37°C, and dialyzed against NET-N(S) buffer as described above. Dialyzed products were stored at -80°C. An aliquot of 1 μg of each purified protein was analyzed for purity by 12.5% SDS-PAGE and Coomassie staining.

### *In vitro* SUMOylation assay (IVSA)

To modify 425 nM His_6_-hLa IVSA was carried out by using 89 nM GST-SAE1, 80 nM GST-SAE2, 0.4 μM His_6_-Ubc9 and 7.3 μM His_6_-SUMO-1/-2/-3GG or His_6_-SUMO-1/-2/-3GA in NET-N(S) buffer (50 mM Tris-HCl, 100 mM NaCl, 0.1% Nonidet P-40, 10% glycerol, pH 8.0) containing 1 x ATP mix (10x ATP-Mix:100 mM creatine phosphate, 20 mM ATP, 50 mM MgCl_2_), 1 U creatine kinase, and 1x complete protease inhibitor mix. Reactions were incubated in a total volume of 25 μl for 2 h at 30°C and either precipitated with 250 μl 100% acetone overnight for Western Blot analysis or stored at 4°C for functional assays. The pellets were twice washed with 250 μl 80% acetone and then air-dried. The pellet was solved in 20 μl SDS loading buffer, heated for 10 min at 95°C, resolved on a 7.5% SDS-PAGE minigel, and proteins were then transferred to NitroPure nitrocellulose membranes (0.45 micron; GE Water and Processing Technologies). Membranes were blocked in 2% BSA (Fraction V, EMD) in wash buffer (10 mM Tris-HCl, 0.1 M NaCl, 0.09% Tween 20 (pH 7.5), incubated a 1:500 mouse monoclonal anti-La3B9 antibody (kind gift of M. Bachmann, [43, 44]) or 1:4,000 rabbit polyclonal anti-SUMO-1 (C-terminus) or 1:4,000 anti-SUMO-2/-3 (N-terminus) antibody (both Enzo Life Sciences). Proteins were detected after incubation with 1:20,000 diluted secondary HRP-mouse/rabbit IgG (Dianova, Hamburg, Germany) using SuperSignal West Pico Chemiluminescent Substrate (Thermo Fisher).

### Non-radioactive RNA electromobility shift assays (EMSAs)

RNA oligonucleotides representing the TOP elements of rat L22 and L37 were synthesized with Cy3 fluorescent dye label at the 3’-end by Integrated DNA Technologies (Coralville, USA). The following sequences were used: L22 (NM_031104), 5'-CUCCUCUGCCGCCAUGGCUCCUGUGAAAAAGCUUGUG-3'; and L37 (NM_031106) 5'- UUCCGGUCUCUUUGGCCUCGCCGGUAGAAGCAAGAUG-3'. The non-radioactive EMSA for CCND1 was performed as described elsewhere [42]. Native 10% polyacrylamide gels for EMSA were pre-run in TBE buffer (45 mM Tris, 45 mM boric acid, 1 mM EDTA) at 140 V for 40 min.

For La:RNA-binding reactions the sumoylation buffer (NET-N(S)) was exchanged to 2x RNA-binding buffer (3 mM MgCl_2_, 300 mM NaCl, 1 mM EDTA, 0.1% Nonidet P-40) by loading IVSA reaction onto centrifugal filter tubes (modified polyethersulfone membrane, MWCO 10 kDa (VWR)). Filter tubes were spun for 10 min at 8,000 x g at 4°C, 2x binding buffer (three times the volume of the IVSA reaction) was added and centrifuged. This procedure was repeated three times. The final concentration was stopped after the original IVSA volume was reached. RNA-oligos (884 nM) were re-annealed for 5 min at 85°C in annealing buffer (10 mM Tris/HCl (pH 7.4), 40 mM NaCl, 0.2 mM EDTA) and placed on ice for 2 min. For the binding reaction, 22 nM (1: 40 dilution) of RNA oligonucleotide was incubated in 1x binding buffer with hLa in a final volume of 40 μl. Binding was allowed for 10 min at room temperature, samples (without loading buffer) were resolved on the pre-run gel for 30 min at 240 V followed by 2 h at 160 V. After electrophoresis gels were wrapped in cellophane foil and scanned on a Typhoon imager (GE Life Sciences) and quantified using ImageQuant TL software (GE Life Sciences). The value of unmodified La at 27 nM was set to 1000 arbitrary units; all other data were normalized against it. The significance of the binding of S-1:La and control La to L37 and L22 RNA oligonucleotides was calculated using an unpaired two-tailed t-test (Prism 4, GraphPad Software, Inc.)

### Identification of SUMO-acceptor sites by mass spectrometry

The *in vitro* SUMOylation assays were performed using 1μg of SUMO-Activating Enzyme 1 (Aos1/Uba2) (human recombinant), 4μg of Ubiquitin-Conjugating Enzyme UBC9 (SUMO E2), untagged (human, recombinant), 4μg of SUMO-3 His6-tagged (human recombinant) in SUMOylation Buffer containing Mg-ATP 0.01M all reagents obtained from a SUMOylation kit (BIOMOL International). Either 1μg of La protein or 4μg of RanGAP1 (positive control) were tested according to the Kit’s manufacturer’s protocols.

LC-MS/MS analyses were performed on a nano-LC 2D pump (Eksigent) coupled to a LTQ-Orbitrap Velos mass spectrometer via a nanoelectrospray ion source (Thermo Fisher Scientific). Peptides were loaded on an Optiguard SCX trap column (5 μm particle, 300Å, 0.5 ID x 23 mm, Optimize Technologies) and eluted on a 360 *μ*m ID x 4 mm, C_18_ trap column prior to separation on a custom-made 150 μm ID x 10 cm nano-LC column (Jupiter C_18_, 3 μm, 300 Å, Phenomenex). Tryptic digests were loaded on the SCX trap and sequentially eluted using salt plugs of 0, 250, 500, 750 mM, 1 and 2 M ammonium acetate, pH 3.5. Peptides were separated on the analytical column using a linear gradient of 5–40% acetonitrile (0.2% formic acid) in 53 min with a flow rate of 600 nL/min. The mass spectrometer was operated in data-dependent mode to switch automatically between survey MS and MS/MS acquisitions. The conventional MS spectra (survey scan) were acquired in the Orbitrap at a resolution of 60 000 for *m*/*z* 400 after the accumulation of 10^6^ ions in the linear ion trap. Mass calibration used a lock mass from ambient air [protonated (Si(CH_3_)_2_O))_6_; m/z 445.120029], and provided mass accuracy within 5 ppm for precursor and fragment ion mass measurements. MS/MS spectra were acquired in HCD activation mode using an isolation window of 2 Da. Precursor ions were accumulated to a target value of 30000 with a maximum injection time of 100 ms and fragment ions were transferred to the Orbitrap analyzer operating at a resolution of 15000 at *m/z* 400. The dynamic exclusion of previously acquired precursor ions was enabled (repeat count 1, repeat duration: 30 s; exclusion duration 45 s).

MS data were acquired using the Xcalibur software (version 2.1 build1139). Peak lists were generated using Mascot distiller (version 2.3.2.0, Matrix science) and MS/MS spectra were searched against the IPI human database containing 75429 forward sequences (version 3.54, released Jan 2009) using Mascot (version 2.3.2, Matrix Science) with a mass tolerance of 10 ppm for precursor ions and 0.05 Da for fragments. The number of allowed missed cleavage sites for trypsin was set to 3, and oxidation (M), deamidation (NQ), carbamidomethylation (C) and SUMOylation (K) (GGTQN: SUMO3) were selected as variable modifications. A software application was developed to search mascot generic files (mgf) for specific SUMO3 fragment ions (e.g. *m/z* 132.0768, 226.0822, 243.1088, 344.1565, 401.1779; and neutral losses of SUMO3 remnant) to produce a mgf file containing only MS/MS spectra of potential SUMOylated peptide candidates. SUMO fragment ions were removed from the corresponding mgf files and searched again using Mascot as indicated above [45]. Manual inspection of all MS/MS spectra for modified peptides was performed to validate assignments.

### Cell culture

The HEK293 cells were purchased from ATCC (Manassas). The cells were cultured in advance DMEM supplement with 2mM L-glutamine (Life Technologies) and 9% FBS (Atlanta Biologicals). The stable cells lines were generated by transfecting cells with gfp, gfp-LaWT and gfp-La K41/200R using FuGENE HD Transfection Reagent (Promega). The stable transfectants were established by neomycin selection and gfp positive cells were enriched by gfp-specific cell sorting and stored in liquid nitrogen. Three independent cell clones were pooled for the experiments.

### RNA immunoprecipitation (RIP) and quantitative PCR

Cells were harvested at 60–80% confluence, washed with ice-cold PBS and lysed with lysis buffer (20 mM Tris-HCl, pH 7.4, 150 mM NaCl, 1% IGEPAL CA-630, 10% glycerol, 1 mM EDTA, 50mM NaF, and 1 mM DTT) supplemented with RNase inhibitors and protease inhibitors. The cleared lysate was incubated with gfp-magnetic beads (MBL International) overnight on the orbital rotor at 4°C. The beads were washed four times with wash buffer I (50 mM Tris-HCl, pH 7.4, 300 mM NaCl, 0.05% IGEPAL CA-630, 20 mM EDTA, 1 mM DTT, and 1 mM MgCl_2_) and three times with wash buffer II (50 mM Tris-HCl, pH 7.4, 300 mM NaCl, 0.05% IGEPAL CA-630, 20 mM EDTA, 1 mM DTT, 1 mM MgCl_2_ and 1 M urea). Control experiments were performed with gfp-expressing cells to assess the background, no or minor amounts of RNA were detected in the gfp-only pellet, suggesting stringent washing conditions. The RNA was isolated and reverse transcribed by RT^2^ First Strand Synthesis Kit (Qiagen). The input and RIP cDNA samples were diluted 1:10 and 1:100 with water respectively and quantitative PCR (qPCR) was performed on the Biorad iCycler-iQ or Biorad CFX384 PCR system using the RT^2^ SYBR Green Fluor qPCR Mastermix (Qiagen) and qPCR primers (Qiagen): CCND1 (PPH00128F), L37 (QT00014105) and L22 (QT00079982). For each primer set, the standard plot was constructed by using known concentration of template in the qPCR analysis. The enrichment of a specific RNA in the IP pellet was determined by calculating the percentage of the respective mRNA in the pellet versus the input material. The enrichment between gfpLaWT and gfpLaK41/200R was compared using the formula: [La K41/200R (RIP/input) / WT (RIP/input)] X 100, and represented as enrichment (% La WT). Prior to qPCR, immunoblot analysis was performed using anti-gfp antibodies (Roche) to assess La pull-down efficiency in the RIP assay.

## Results

### *In vitro* SUMOylation assay for recombinant human La

**Fig 1:**
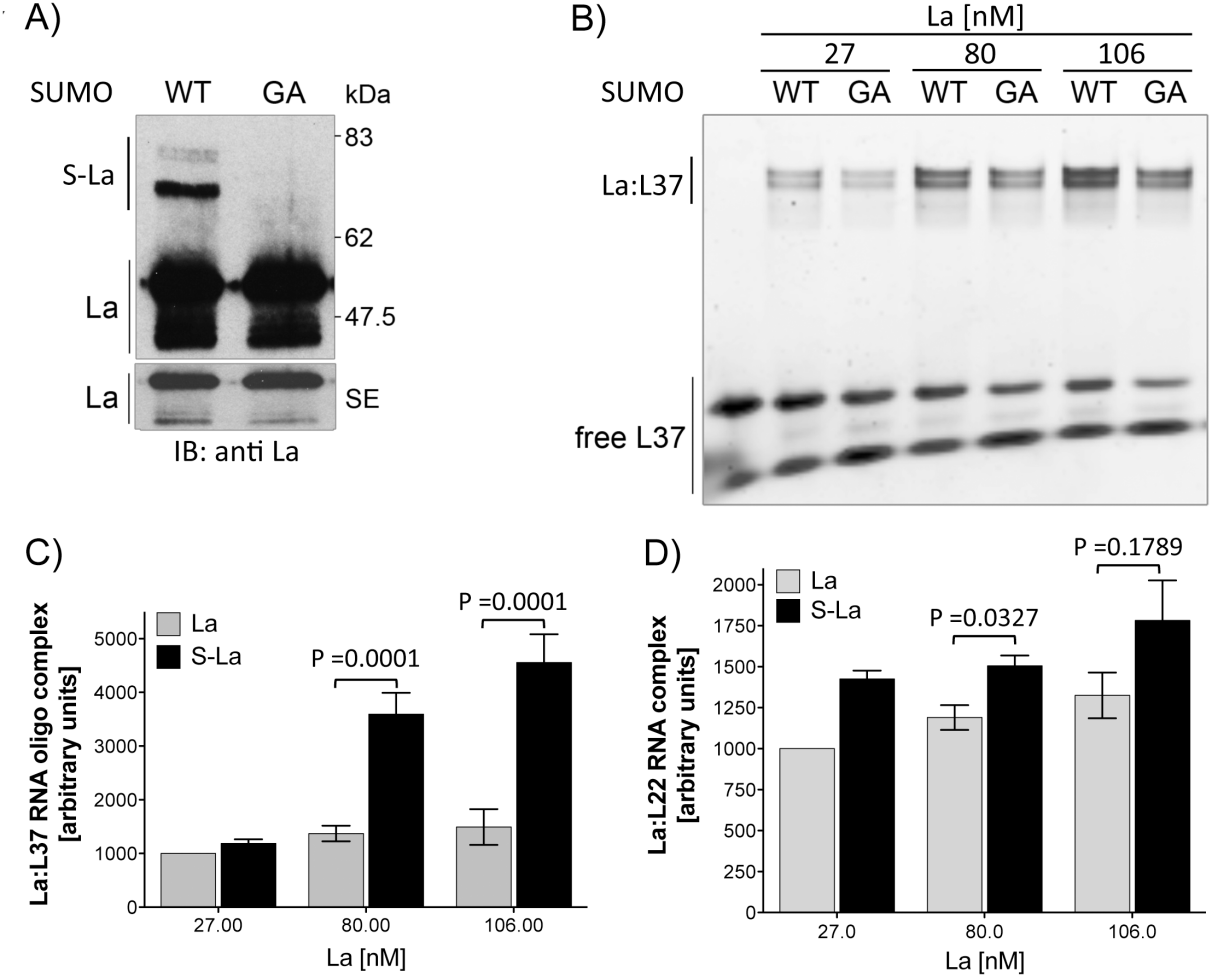
Modification of the La protein with SUMO facilitates binding to L37 and L22 RNA. A) Representative immunoblot analysis of a sumoylation reaction used for electrophoretic mobility shift assay (EMSA). The sumoylated La bands are detectable between 62 and 83 kDa (S-La). The short exposure (SE) shows equal amounts of La. After sumoylation the buffer was exchanged to the optimal RNA-binding buffer for La. B) RNA-binding of native and sumoylated La to 5’-terminal oligopyrimidine (TOP) elements of L37 and L22 RNA was studied by native EMSAs using fluorescence-labeled RNA oligonucleotides. Representative fluorescence-based EMSA of La-binding to L37 RNA are shown. After performing in vitro sumoylation assay, 27, 80 and 106 nM La containing SUMO-WT or SUMO-GA was titrated against 22 nM of L37 RNA. C) Quantification of EMSAs shows that significantly more L37 RNA was shifted by sumoylated La (S-La, black bar) when compared to the control La (La, grey bar). The value of native La at 27 nM was set to 1000 arbitrary units, all other data were normalized against it (* = *P* < 0.0001, n = 10). D) Quantification of EMSAs shows that significantly more L22 RNA was shifted by sumoylated La (S-La, black bar) when compared to the control La (La, grey bar). The value of unmodified La at 27 nM was set to 1000 arbitrary units; all other data were normalized against it (* = *P* <0.0327, n = 3). The *p*-value was determined from ten (L37) or 3 (L22) independent experiments using in Prism5 software and applying the two-tailed paired Student’s t-test.

It has been previously shown that the RNA-binding protein La can be modified by SUMO-1 and SUMO-2 [23, 34], raising the question of whether sumoylation alters the function of La. La has been shown to bind to several different mRNAs, thus we focused on La’s RNA binding activity. To address this question, we established an *in vitro* SUMOylation assay (IVSA) for the La protein, using His-tagged recombinant human La, His-tagged wild-type SUMO-1 (SUMO-WT), the GST-tagged SUMO activating enzymes SAE1 and SAE2, and the His-tagged conjugating enzyme Ubc9. The 3’-terminal di-glycine motif of SUMO is essential for the ligation to the target protein and mutation of the terminal G to an A prevents the ligation to the target protein [41, 46]. In parallel reaction, SUMO-WT and SUMO-GA mutants were used. The SUMO-GA mutants were used to control for non-specific modifications to recombinant La. Since SUMO-GA mutants were not ligated, we referred to the La in those reactions as native La. Nickel column purified proteins were used to establish an *in vitro* IVSA for the RNA-binding protein La. To detect sumoylated La, we analyzed IVSA samples by immunoblot analysis using La-specific antibody La 3-B9 (Fig 1A, [43, 44]). We found that only a minor fraction of recombinant La was modified by SUMO and that La can be sumoylated (S-La) at more than one site. The predominant band at about 70 kDa might represent La modified by two SUMO molecules (11 kDa per SUMO). We hypothesize that the higher band below 83 kDa is a triple SUMO-modified La protein (Fig 1A, S1A Fig). As expected, our sumoylation control SUMO-GA did not conjugate with recombinant La, supporting the specificity of the IVSA. We observed that sumoylation was slightly more efficient at 30°C than at 37°C (S1B Fig) and that the ATP-dependent sumoylation process was strongly impaired in the presence of non-hydrolysable γATP (S1C Fig). Furthermore, we found that SUMO conjugation is not stimulated by the ligases BP2**Δ**FG and IR1+M [47] (S2 Fig) and that the SUMO-1:Ubc9 ratio of 1:10 is optimal for sumoylation of La (data not shown). Although we optimized the sumoylation of La, we observed that only a small fraction of La was sumoylated under our conditions (S3A Fig). To quantify the sumoylation of La, we acetone-precipitated the La protein and analyzed the reaction by immunoblotting and quantified the reaction by recording the chemiluminescence signal over time. Analysis of four independent sumoylation reactions revealed that approximately 5% of the input La was sumoylated (S3B Fig). In summary, *in vitro* sumoylation of La leads to two La-specific bands—referred to as S-La—running between the molecular weight markers 62 and 83 kDa (Fig 1A).

Although a target protein is often modified specifically by either SUMO-1 or SUMO-2/-3 *in vivo*, it has been reported that many substrates can be sumoylated by all three SUMO paralogs *in vitro* [48]. Hence, we tested whether all three SUMO paralogs can be conjugated to La. As shown (S4 Fig) wild-type (WT) SUMO-1, -2 or -3, but not the mutant proteins SUMO-1-GA, -2-GA or -3-GA were ligated with comparable efficiency to recombinant La. Earlier studies demonstrated that endogenous SUMO-1 present in rat neurons modifies endogenous La and that transfected SUMO-2, but not SUMO-3 was able to modify gfp-tagged La in rat neurons [23]. This suggests that the neuronal cells compartmentalize the different SUMO paralogs, only giving La access to endogenous SUMO-1 modification in the axonal compartment. A recent screen for sumoylated proteins after heat shock showed that endogenous La was modified by SUMO-2 in the human cervical cancer cell line HeLa [34]. Thus, it is likely that SUMO-type specific modifications of La occur in different cell types.

### Binding of La to different TOP elements

Next, we aimed to test whether the sumoylation of La alters the binding affinity of La for mRNAs. For this, we used short RNA oligonucleotides corresponding to the TOP elements of L37 and L22 mRNAs. Binding of La protein to TOP mRNAs has been established *in vitro* and in cells [18–21, 49]. We reasoned that these transcripts could be exposed to sumoylated La, since both of these TOP mRNAs localize to the axonal compartment of rat sensory neurons where La has been shown to undergo sumoylation in these primary neurons [23, 50].

RNA oligonucleotides representing the TOP elements of rat L22 and L37 were applied to establish the optimal conditions for binding of La to the L37 and L22 RNA. Recombinant La concentrations were titrated over 6 to 700 nM, and binding was monitored by applying electrophoretic mobility shift assays (EMSA) (S5 Fig). RNA-binding of La was quantified by recording the Cy3 fluorescence intensity of the free versus protein-bound RNA. Most of the L37 and L22 RNA (22 nM) were bound by La at a concentration between 200 and 400 nM La, suggesting that a large fraction of recombinant La protein is not binding competent as also described earlier [51].

Next, we directly compared the RNA-binding activity of SUMO-modified La (S-La) and native La to L37 and L22 RNA. As shown by representative immunoblot (Fig 1A), the sumoylation efficiency and concentration of La was validated each time. We used 27, 80, and 106 nM of total La (SUMO-modified and native) and 22 nM of L37 or L22 RNA for the binding reaction. The representative EMSA analysis (Fig 1B) and quantification of a series of those experiments demonstrate that SUMO-modified La binds significantly more L37 RNA than native La (Fig 1C). Although a similar trend was observed with L22 RNA, the increase in binding by SUMO-modified La was only significant at La concentration of 80 nM (Fig 1D). We conclude that SUMO-modified La binds especially L37 RNA more efficiently than native La. Furthermore, we speculate that modification of La by SUMO induces conformational changes in La more favorable for RNA-binding.

### The human La protein is sumoylated at lysine 200 and 208

**Fig 2:**
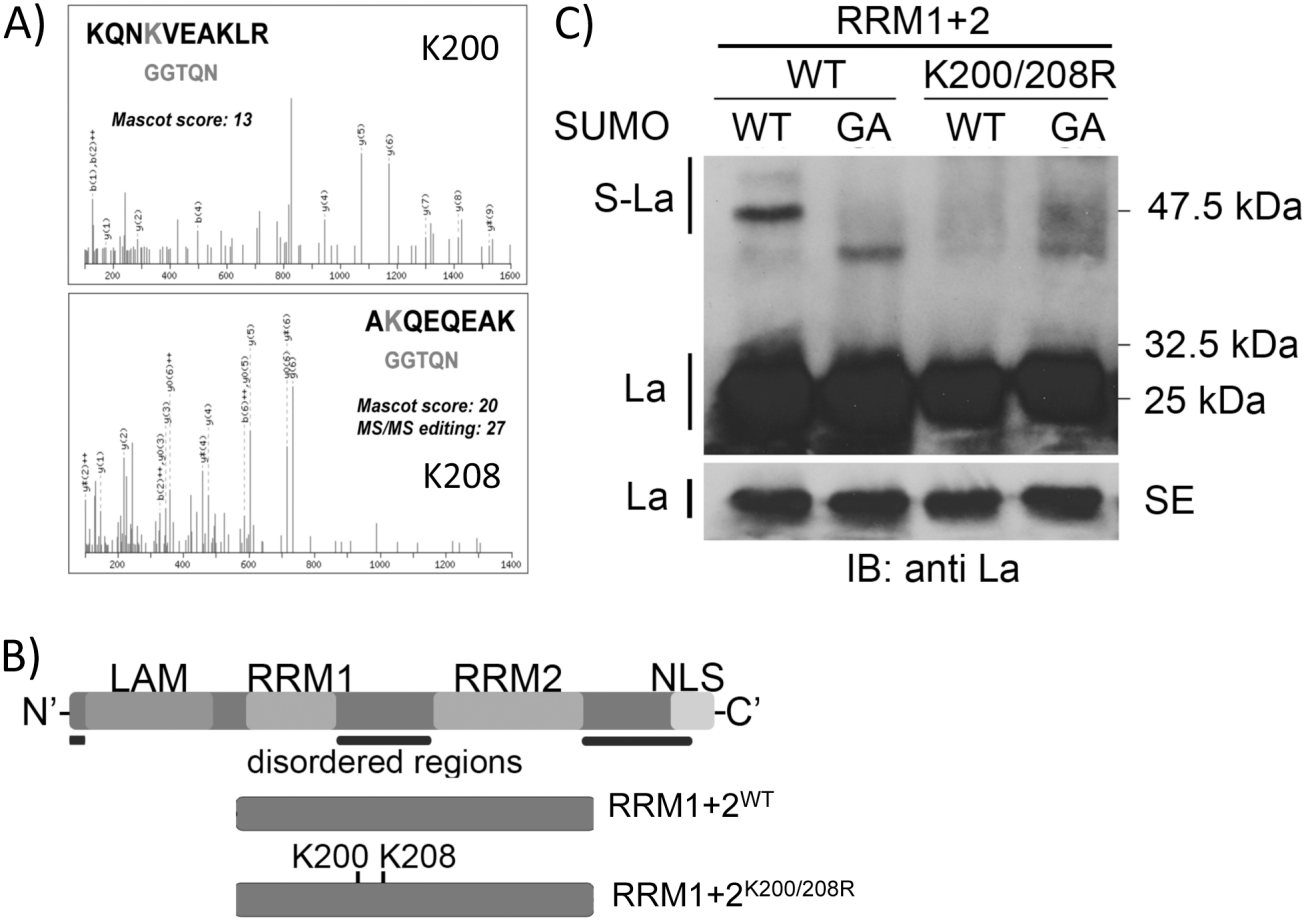
Identification of lysine 200 and 208 as SUMO-acceptor sites. A) Identification of lysine 200 (KQNK_200_VEAKLR) and 208 (AK_208_QEQEAK) as SUMO-acceptor sites in recombinant La by mass spectrometry. B) Schematic view of the La domains and position of the two SUMO-acceptor sites (K200 and K208). The RRM1+2 represents a minimal RNA-binding competent La mutant used in these studies. LAM = La motif, RRM = RNA Recognition Motif, and NLS = Nuclear Localization Signal. C) Lysine 200 and 208 are sumoylation sites of human La *in vitro*. In order to study the effect of SUMO-modification on the La protein, the main sumoylation sites were confirmed by IVSA by mutating the two sites in RRM1+2^K200/208R^. Recombinant proteins were sumoylated with SUMO-WT and SUMO-GA, acetone precipitated overnight, and analyzed by immunoblotting (IB) for La. Sumoylated (S-La) and native RRM1+2 (La) is indicated on the left. The molecular weights are indicated in kDa on the right. SE = short exposure.

To identify the SUMO-acceptor sites in recombinant La, we applied mass spectrometry to the *in vitro* sumoylation assay modified La protein [45]. Lysine residues 200 and 208 were identified as the main SUMO-acceptors sites in the recombinant La protein (Fig 2A). Interestingly, both SUMO-acceptor sites are comprised in SUMO consensus motifs located in the intrinsically disorder linker region between RNA Recognition Motif 1 (RRM1) and RRM2 [52] (Fig 2B) and lysine 208 has also been identified also as a SUMO-acceptor site in rat axons [23]. We also identified other non-consensus SUMO-acceptor sites at lysine residues 344, 354, and 363 though their abundances were significantly lower than those located in the linker region. To clearly demonstrate that lysine residues 200 and 208 are SUMO-acceptor sites and to test whether sumoylation alters the RNA-binding activity of La, lysine 200 and 208 (K200/208) were both mutated to arginine residues (200R/208R) in the minimal RNA-binding competent La fragment spanning amino acids 114 to 326 [42], referred to as RRM1+2^K200R/208R^ (Fig 2B). RRM1+2^WT^ contains both RRM1 and RRM2 and binds RNA efficiently [42]. RRM1+2^WT^ and RRM1+2^K200R/208R^ were expressed, purified, and subjected to *in vitro* sumoylation. In parallel reactions, SUMO-WT and SUMO-GA mutants were used. The SUMO-GA mutants were used to control for non-specific modifications to recombinant RRM1+2^WT^ or RRM1+2^K200R/208R^ and we refer to those proteins as native RRM1+2^WT^ or native RRM1+2^K200R/208R^. As shown, RRM1+2^WT^ was efficiently sumoylated, whereas RRM1+2^K200R/208R^ was not modified (Fig 2C). Interestingly, testing the single SUMO-acceptor site mutant RRM1+2^K208R^, we showed that this mutant was strongly sumoylated, suggesting that K200 is the main SUMO-acceptor site in recombinant RRM1+2 (S6A Fig). In presence of recombinant SUMO-GA a minor 45 kDa band was observed. At this point we do not know the nature of this band and why this band appears only if we perform *in vitro* sumoylation with La-RRM1+2 substrate and not with full-length La.

**Fig 3:**
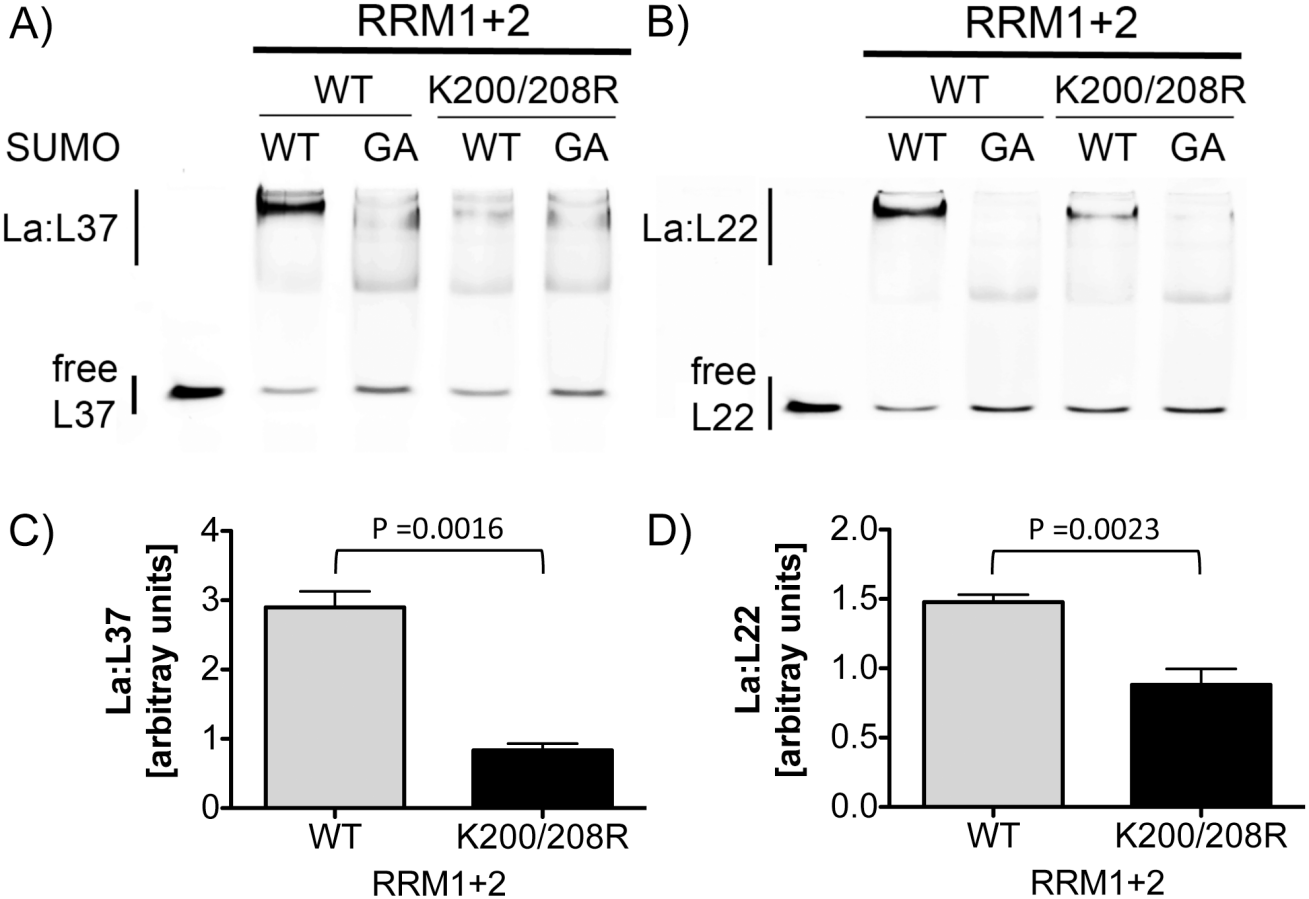
Sumoylation facilitates La binding to L37 and L22 RNA. A) Representative fluorescence-based EMSA showing binding of sumoylated RRM1+2^WT^ and RRM1+2^K200/208R^ to L37 and L22 RNA, and binding of control-treated proteins applying conjugation-incompetent SUMO-GA. For EMSAs, La protein from *in vitro* sumoylation assays containing SUMO-WT or SUMO-GA and L37 or L22 RNA was used. B) Quantification of EMSAs shows that significantly more L37 RNA (** = *p* < 0.0016, n = 5) or L22 RNA (** = p < 0.0023, n = 5) were shifted by samples containing sumoylated RRM1+2^WT^ (grey bar) when compared the double mutant RRM1+2^K200/208R^ (black bar).

Next, we compared the RNA-binding activity of RRM1+2^WT^ and RRM1+2^K200R/208R^. Recombinant RRM1+2^WT^ and RRM1+2^K200R/208R^ were purified, sumoylated, and subsequently tested for binding to L37 and L22 RNA. As shown by representative EMSAs (Fig 3A and 3B) and quantification (Fig 3C and 3D), only SUMO-modified RRM1+2^WT^ and not native RRM1+2^WT^ led to a strong La:L37 or La:L22 RNA complex. Sumoylation of RRM1+2^K200R/208R^ did not enhance binding of RRM1+2^K200R/208R^ to either L37 or L22 RNA and bound the RNA only weakly as observed with native RRM1+2^K200R/208R^. These results confirm that lysine 200 and 208 are SUMO-acceptor sites supporting binding of La to L37 and L22 RNA.

**Fig 4:**
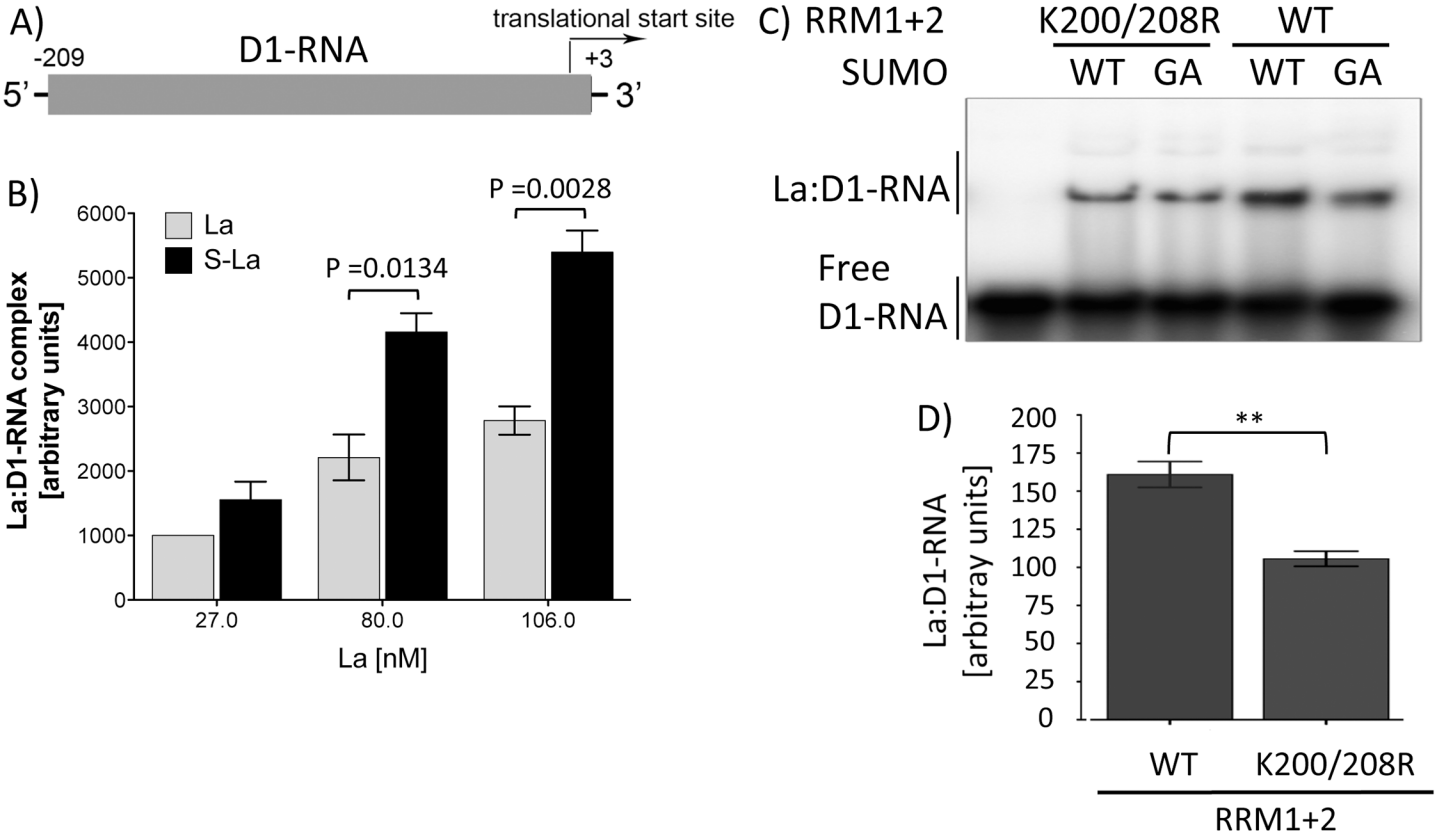
Sumoylation facilitates La binding to the 5’-UTR of CCND1 mRNA. A) Scheme of the *in vitro* transcript representing the 5’-UTR of CCND1 RNA (D1-RNA). B) Quantification of EMSAs shows that significantly more D1-RNA was shifted by in vitro sumoylated samples containing sumoylated La (black bar, using SUMO-WT) when compared to the control La (grey bar, using SUMO-GA). The value of native La at 27 nM was set to 1000 arbitrary units; all other data were normalized against it (n = 4). C) Representative EMSA showing binding of sumoylated RRM1+2^WT^ and RRM1+2^K200R/208R^ to ^32^P-labeled D1-RNA. For EMSA, the La from *in vitro* sumoylation assays in presence of SUMO-WT or reaction-incompetent SUMO-GA was used to test binding to D1-RNA. D) Quantification of EMSAs shows that significantly more D1-RNA (n = 3) was shifted by samples containing sumoylated RRM1+2^WT^ (WT) when compared to the double mutant RRM1+2^K200R/208R^ (K200R/208R).

The L37 and L22 RNA are short oligonucleotides with weak secondary structures compared to the La-binding site in CCND1 RNA [42]. Hence, we asked the question whether sumoylation also alters the binding of La to the 5’-UTR (212 nts in length, referred to as D1-RNA) of CCND1 mRNA (Fig 4A). Binding of recombinant La to *in vitro* transcribed and ^32^P-labeled D1-RNA has recently been shown [42]). Quantification of EMSAs performed with native or SUMO-modified La demonstrates that sumoylated La binds more D1-RNA (Fig 4B), as we have shown before for L22 and L37 RNA. Furthermore, as shown by EMSA performed with L22 and L37 RNA, only sumoylation of RRM1+2^WT^ (Fig 4C, right two lanes) led to efficient binding, whereas RRM1+2^K200R/208R^ (Fig 4C, left two lanes) binding to D1-RNA was significantly weaker (Fig 4C and 4D). The results support the assumption that sumoylation of La facilitates the binding of short RNA oligonucleotides as well as longer structured RNA elements as present in the 5’-UTR of CCND1 mRNA.

### Sumoylation regulates La association with mRNAs in cells

Recently, three potential SUMO-acceptor sites in La were reported lysine 41, 185, and 208 and it has been shown that lysine 41 is the main SUMO-acceptor site in rat axons [23]. To test whether those sites are also sumoylated *in vitro* we expressed recombinant human La with a mutation in lysine 41 (La^K41R^) and a triple mutant of La with lysine to arginine mutations in K41, K185, and K208, referred to as La^K41R/K185R/K208R^, and tested those for *in vitro* sumoylation. We found that both La^K41R^ and La^K41R/185R/208R^ were still modified by SUMO *in vitro* (S6B and S6C Fig), however only a single S-La band was detectable with La^K41/185/208R^, suggesting that at least one functional SUMO-acceptor site (e.g. K41) was deleted and that a major acceptor site (e.g. K200) was still available. Because K41 was found as a major SUMO-acceptor site in rat axons, we have to consider that *in vitro* sumoylation might not precisely reflect the sumoylation events occurring in cells. In addition, we have to assume that in different cell types or cell lines different SUMO-acceptor sites may be used, as supported by a recent report demonstrating sumoylation of the La protein at lysine 35, 86, and 400 in HeLa cells [35, 36] and lysine 76, 86, 105 and 116 in HEK293 cells [37].

**Fig 5:**
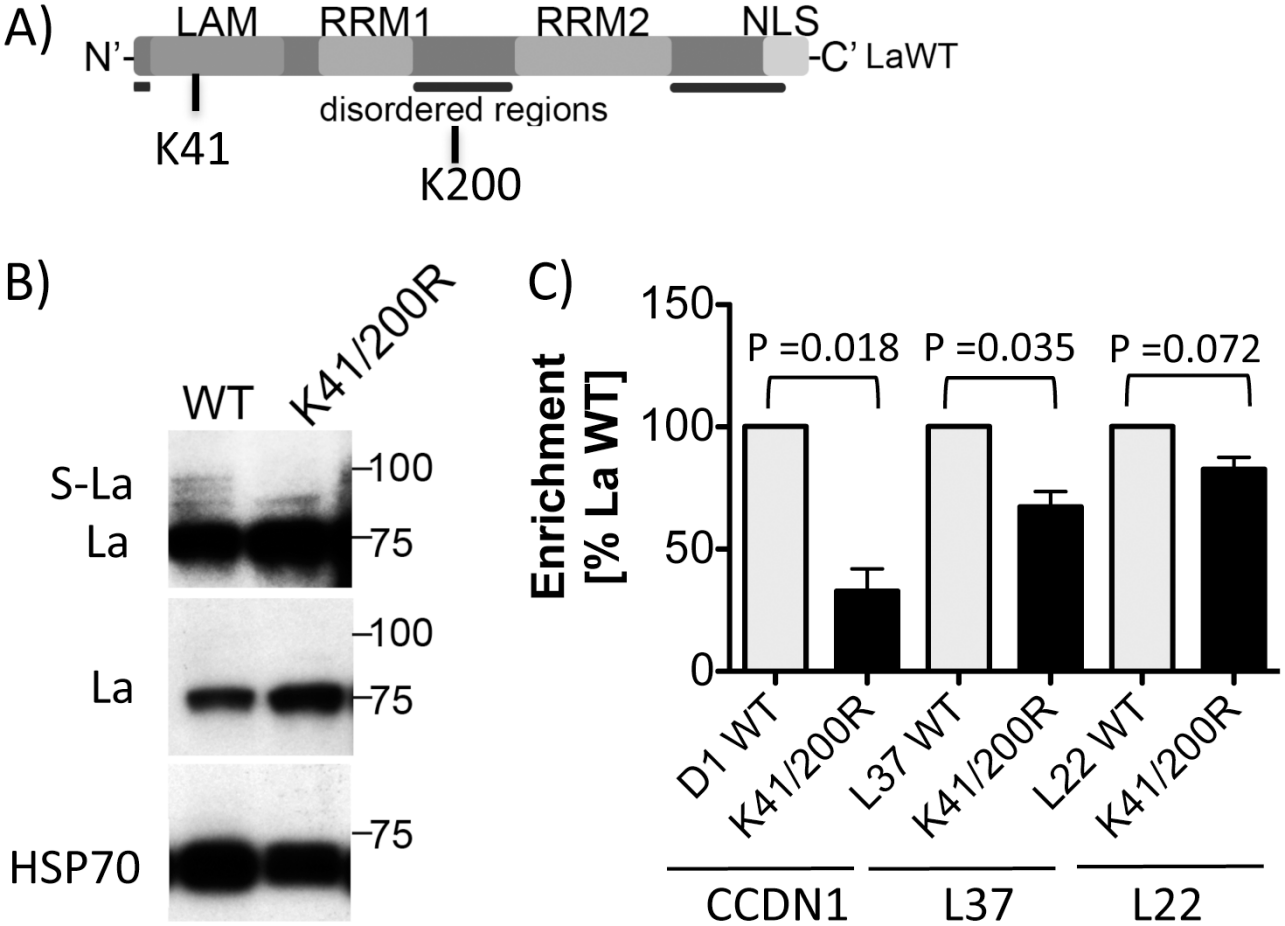
SUMO-acceptor site mutation K41/200R reduces RNA-binding of La in HEK293 cells. A) Schematic of La showing K41 and K200 SUMO-acceptor sites in LAM (light grey bar) and RRM1+2 (dark grey bar), respectively; LAM = La motif, RRM = RNA Recognition Motif, NLS = Nuclear Localization Signal. B) Representative immunoblot analysis of La in gfp-tagged La^WT^ and La^K41R/200R^ expressing cells; S-La indicates three high molecular weight bands. HSP70 was used as loading control. C) RNA Immunoprecipitation (RIP): RT-qPCR analysis of CCND1, L37, L22 transcripts associated with gfp-tagged La^WT^ and La^K41R/200R^ in HEK293 cells. The percentage of enrichment of mRNA in the La^WT^ RIP pellet compared to the input material was set as 100%. Compared to La^WT^, significantly less CCND1 mRNA and L37 mRNA was enriched in the La^K41R/200R^ RIP pellet (CCND1 = *p* <0.018 (n = 3), L37 = *p* <0.035 (n = 3). The enrichment of L22 mRNA was not significantly reduced L22 = *p* <0.072 (n = 3). The *p*-value was determined from ≥3 independent experiments using in Prism5 software and applying the two-tailed paired Student’s t-test.

To test whether sumoylation facilitates the binding of La to mRNAs in cells, we created gfp-tagged La expression vectors to perform RNA immunoprecipitation using the gfp-tag for pull-down. Because lysine 41 was the main acceptor site in rat axons [23] and lysine 200 appeared to be the main acceptor site *in vitro*, we created the double mutant La^K41R/200R^ (Fig 5A) and expressed this mutant and La wildtype (La^WT^) with a gfp-tag in HEK293 cells. First, we aimed to demonstrate sumoylation of La^WT^ and La^K41R/200R^. We found that a small fraction of La^WT^ was modified, indicative by three faint, higher molecular bands (S-La) in addition to the major La band. Two of those bands were not detectable with La^K41R/200R^ (Fig 5B), suggesting that only low amounts of La are sumoylated at a given time as also observed for other sumoylated proteins in different cells and *in vitro* [30–33]. This finding suggests that mutation of K41 and 200 impaired sumoylation of La in HEK293 cells. Since our *in vitro* data suggested that sumoylation enhances the ability of La to interact with RNA, we performed RNA immunoprecipitations using HEK293 cells stably expressing gfp, gfp-tagged La^WT^ or La^K41R/200R^ and gfp-specific antibodies to compare the enrichment of mRNAs in the pellet of La^WT^ or La^K41R/200R^ cells. RNA was extracted from the input material and immunoprecipitation (IP) pellets, reverse transcribed and analyzed by quantitative PCR using standard curves for CCND1, L22, and L37. Recently, it has been shown that CCND1 [11] and L37 mRNAs [21] are associated with La in HeLa and HEK293 cells, respectively. Note; very low RNA amounts were detected in the gfp IP pellets, suggesting that the gfp-tag is not accounting for mRNA pull-down. The percentage of a specific RNA in the IP pellet versus the input of La^WT^ cells was set as 100% and then compared to the percentage of the same mRNA in the IP pellet versus the input of La^K41R/200R^ cells. By comparing the enrichment of CCND1, L22 or L37 mRNA in La^WT^ and La^K41R/200R^ cells, we found that significantly less CCND1 and L37 mRNA was associated with La^K41R/200R^, suggesting that the binding of La to CCND1 and L37 mRNAs was impaired in La^K41R/200R^ expressing cells. Further, the binding of L22 mRNA by La^K41R/200R^ was also reduced, but not significantly. The RNA immunoprecipitation data suggest that sumoylation facilitates RNA-binding of La in cells. Since CK2-dependent La phosphorylation is known to impair L37 RNA binding [21], we tested whether the S366 phosphorylation status is different in La^WT^ and La^K41R/200R^ cells. No difference in CK2-dependent La phosphorylation was observed between La^WT^ and La^K41R/200R^ cells, suggesting that CK2-dependent phosphorylation of La is not coupled to its sumoylation (VK and TH, unpublished data).

## Discussion

Herein we demonstrate that recombinant human La protein can be sumoylated at lysine residues 200, 208, and that sumoylation facilitates binding of La to small and larger mRNA molecules *in vitro* and in cells. Both SUMO-acceptor sites are located in the intrinsic disordered linker region connecting the two RRMs of human La.

Recently, both RRMs has been shown to be sufficient for binding HBV [38] and CCND1 mRNAs [42] and are required for binding to the HCV RNA [53]. The assumption that both RRMs are acting together [38] to bind selective RNA molecules suggests that folding of the La protein is critical to reach an optimal spatial arrangement of the two RRMs. As the main SUMO-acceptor sites are located between the two RRMs, we expected to observe a negative effect of sumoylation on the RNA-binding activity of La considering that SUMO peptides might lead to steric hindrance of RNA-binding. However, herein we show that sumoylation facilitates RNA-binding by La. We propose a model, in which sumoylation converts improperly folded La protein into RNA-binding competent La or stabilizes the structure of RNA-binding competent La, hence, it will be interesting to study in future experiments, whether sumoylation induces structural changes in La.

In order to begin to establish a functional significance of these findings in a cell-based assay, we performed RNA immunoprecipitation (RIP) experiments to test whether sumoylation-deficient La was associated with fewer RNA molecules. These studies revealed that sumoylation indeed regulates the RNA-binding activity of La in cells. During these studies we experienced major difficulties to document sumoylation of La in HEK293 cells. Because only a minor fraction of La is sumoylated at a given time, it will be important for future studies to identify cell lines in which a higher fraction of La is modified or to identify a condition triggering sumoylation of La such as heat shock in HeLa cells [34].

**Fig 6:**
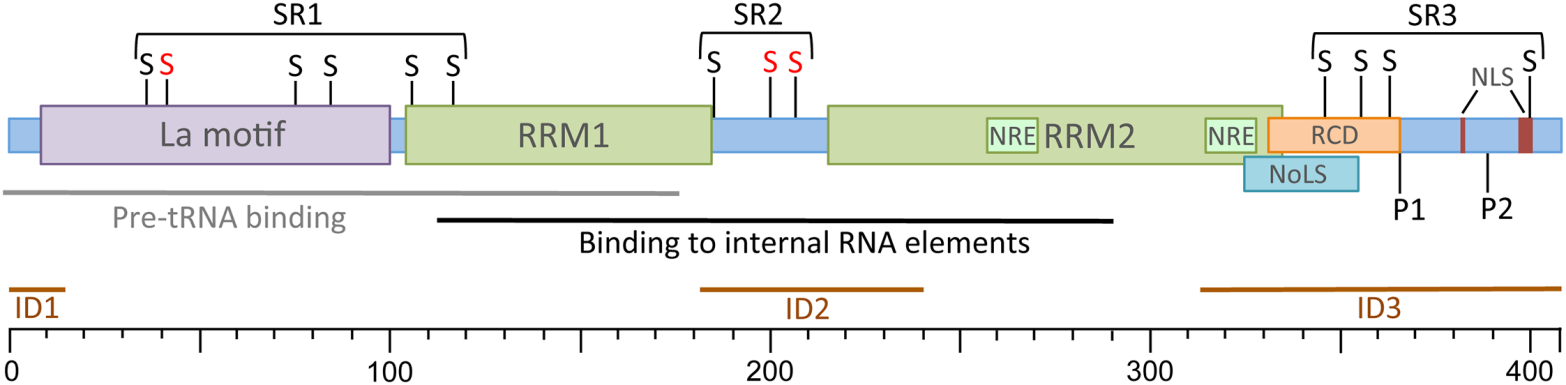
SUMO-acceptor sites in La. SUMO Region = SR; S = SUMO-acceptor site (red = mutated in this study, black = potential SUMO-acceptor sites [23, 35–37]; LAM = La Motif; RRM = RNA Recognition Motif; NRE = Nuclear Retention Element; RCD = RNA Chaperone Domain; NoLS = Nucleolar Localization Sequence; NLS = Nuclear Localization Signal; P1 = CK2 phosphorylation site; P2 = AKT phosphorylation site; ID = internal disordered region.

To our knowledge, so far 13 SUMO-acceptor sites are described for La. In rat axon lysine 41 is the main acceptor site, and lysine 185 might be a potential SUMO-acceptor site [23]. Lysine residues 200 and 208 are major, and 344, 354, and 363 are minor acceptor sites *in vitro* (this study). In addition, lysine 35 and 86 [35], lysine 400 in HeLa cells [36], and lysine 76, 86, 105 and 116 in HEK293 cells [37] were identified as potential SUMO-acceptor sites. As summarized in Fig 6, the SUMO-acceptor sites appear to concentrate in three distinct areas of La: SUMO Region1 (SR1: lysine 35, 41, 76, 86, 105, 116) contains the La motif and the beginning of the RRM1—both domains are engaged in binding of RNA polymerase III transcripts [54]; SUMO Region2 (SR2: lysine 185, 200, 208) in the linker region between RRM1 and RRM2 engaged in binding to cellular and viral mRNA [38, 42]; and SUMO Region3 (SR3: lysine 344, 354, 363) within the RNA chaperone domain (RCD) [42] and Nucleolar Localization Signal (NoLS) [55, 56] near the C-terminus of La. The Nuclear Retention Element (NRE) [57, 58] lies between SR2 and SR3 whereas the CK2 [21, 59] and AKT phosphorylation sites at serine 366 and threonine 389 [42], respectively, are located within SR3. Note that the C-terminal domain is considered to be intrinsically disordered [52], suggesting that sumoylation in SR3 might alter the folding of La at the C-terminal intrinsic disordered region, its phosphorylation status, its RNA chaperone activity, and/or its nucleolar localization. In future studies, it will be of interest to determine whether sumoylation in SR1 alters the processing of RNA polymerase III transcripts, or sumoylation in SR2 influences the translation of mRNAs, and whether sumoylation in SR3 changes the phosphorylation and the sub-nuclear/-cellular distribution of La. Recent work suggests that the overexpression of La in cancerous cells might contribute to tumorigenesis [11, 12, 15, 60, 61], hence it will be interesting to compare the sumoylation status of La in normal and cancerous cells. This is of great interest because recent work proposes that aberrant sumoylation contributes to cancer pathology [28, 62, 63].

An increasing number of RNA-binding proteins have been reported to be sumoylated [64], and sumoylation plays a role at different steps during mRNA processing as summarized elsewhere [24]. As an example the RNA-binding protein nucleolin is sumoylated at lysine residue 294 [65]. Lysine 294 is located in the nuclear localization signal at the end of the N-terminal domain just before the first RRM1 (aa 307 to 383). Although RIP experiments show that mutation of lysine 294 led to a weaker association with GAD45alpah RNA, it is not clear whether sumoylation changes the RNA-binding activity of nucleolin or whether the differential sub-cellular localization of SUMO-deficient and wildtype nucleolin accounts for this result [65]. Furthermore, sumoylation of yeast THO complex subunit *hpr1* is required for mRNA recruitment [66]. In addition, sumoylation of a number of other RNA-binding proteins [64, 67] and proteins involved in RNA processing [68, 69] have been reported. As an example, sumoylation impairs the binding of hnRNPC1 to single-stranded DNA [67], and it negatively regulates the RNA editing activity of ADAR1, suggesting that the affinity for substrates can be affected by this post-translational modification [70]. Furthermore, sumoylation of the translation initiation factor 4E stimulates cap-dependent translation of different mRNAs [71] and regulates the activity of SAM68 in CCND1 translation [72].

In summary, the positive effect of sumoylation on the RNA-binding activity of La might alter its role in RNA processing or its sub-cellular localization and thereby might change a number of central cellular and tumor-promoting processes, such as cell proliferation, invasion, and motility where the function of La has been implicated.

## Supporting Information

S1 Fig The human La protein is modified by SUMO *in vitro*. A) *In vitro* sumoylation assay was carried out with recombinant human La protein, SAE1, SAE2, Ubc9, SUMO-WT or conjugation-defective SUMO-GA at 30°C for 2 hours. Immunoblot analysis shows La-specific bands between 62 and 83 kDa representing sumoylated La (S-La), because those bands were not detectable with conjugation-defective SUMO-GA. B) The efficiency of the *in vitro* sumoylation of recombinant La protein is sensitive to temperature. Sumoylation was carried out in parallel at 30°C and 37°C, aliquots were taken at 0.5, 1, 1.5, and 2 hours. As negative control reactions were performed in parallel with conjugation-defective SUMO-GA. The incubation at 30°C resulted in S-La after an hour (upper blot), whereas the incubation at 37°C results in an overall weaker sumoylation of La after 1.5 hours (lower blot). C) The IVSA of the La protein depends on ATP as an energy donor. The *in vitro* sumoylation assay was performed in the presence and absence of ATP and in the presence of the non-hydrolysable γATP analog. The native La and S-La species are indicated on the left, the molecular weights in kDa on the right. SE = short exposure, IB = immunoblot, S-La = sumoylated La).
(TIF)

S2 Fig RanBP2 ligase activity does not facilitate SUMO-1 modification of La. In order to determine if RanBP2 ligase activity can increase the efficiency of the in vitro sumoylation assay of recombinant human La, two minimal functional recombinant RanBP2 proteins (BP2DFG and IR1+M) were purified and used in the IVSA. The sumoylation assay was carried out for 2 hours at 30°C and samples were analyzed as described. Sumoylated La (S-La) is indicated on the left. Modification of La is observed only when SUMO-WT is used and not in the presence of the conjugation-deficient SUMO-GA mutant. The short exposure (SE) shows similar La levels (lower blot). The molecular weight (kDa) is indicated on the right.
(TIF)

S3 Fig The detection of sumoylated La is improved by acetone precipitation. To allow the analysis of the complete *in vitro* sumoylation assay products and to enhance the immunodetection, samples were precipitated with acetone overnight. A) *In vitro* sumoylation assay was performed and aliquots of different La amounts (50, 125, and 250 ng) were taken and precipitated overnight followed by immunoblot (IB) analysis for La. Sumoylated La (S-La) is indicated on the left. B) Immunoblot quantification of *in vitro* sumoylation assay products revealed that on average only 5% of total La is sumoylated. Chemiluminescence signals of four independent *in vitro* sumoylation assay products were recorded (n = 4) and quantified using the ImageQuant RT ECL instrument and ImageQuant TL software.
(TIF)

S4 Fig La is sumoylated by all three SUMO paralogs *in vitro*. A) Immunoblot (IB) analysis for La shows La-specific bands representing sumoylated La (S-La) in samples with all three SUMO-WT paralogs, but not in conjugation-incompetent SUMO-GA controls. SE = Short Exposure.
(TIF)

S5 Fig Binding of La to two different TOP mRNA elements. To study the binding of La to L37 and L22 RNA, Cy3-labeled RNA-oligonucleotides representing the 5’-terminal 37 nts of rat L37 and L22 were synthesized. For the binding reactions 22 nM of the RNA oligonucleotides and 6.0, 12.5, 25, 50, 100, 200, 300, 400, 500, 600, and 700 nM of recombinant human La was titrated. Fluorescence of Cy3-labeled free RNA and La-bound RNA were detected using a Typhoon Imager. No La protein was added in the first lane.
(TIF)

S6 Fig Additional La mutants tested for SUMOylation. *In vitro* sumoylation assay was carried out for 2 hours at 30°C, reactions were subjected to immunoblot (IB) analysis for La. The following La mutants with lysine to arginine substitutions were tested: A) RRM1+2^K208R^ and RRM1+2^K200R/208R^; B) La^K41R^; C) LaSM123 (triple mutation of K41R/195R/208R). Immunoblot (IB) analysis shows La-specific bands between 62 and 83 kDa (S-La). Those bands were not detectable with reaction-incompetent SUMO-GA.
(TIF)
