# Supplementary figures and images for: SUMO-Modification of the La Protein Facilitates Binding to mRNA In Vitro and in Cells
Source: PLoS One. 2016 May 25;11(5):e0156365. doi: 10.1371/journal.pone.0156365 (PMC4880191; doi:10.1371/journal.pone.0156365)

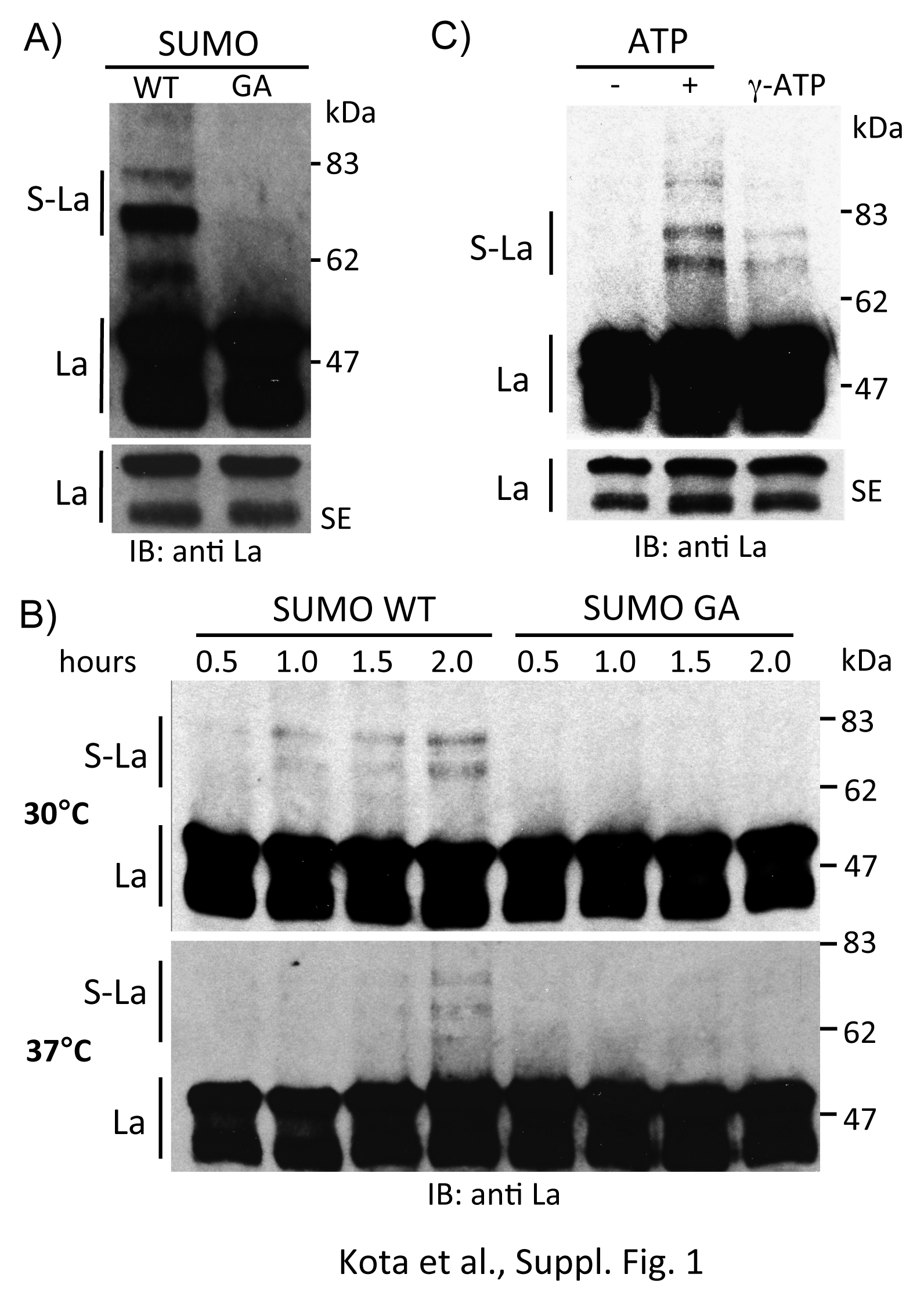

Supplement: S1 Fig — A) In vitro sumoylation assay was carried out with recombinant human La protein, SAE1, SAE2, Ubc9, SUMO-WT or conjugation-defective SUMO-GA at 30°C for 2 hours. Immunoblot analysis shows La-specific bands between 62 and 83 kDa representing sumoylated La (S-La), because those bands were not detectable with conjugation-defective SUMO-GA. B) The efficiency of the in vitro sumoylation of recombinant La protein is sensitive to temperature. Sumoylation was carried out in parallel at 30°C and 37°C, aliquots were taken at 0.5, 1, 1.5, and 2 hours. As negative control reactions were performed in parallel with conjugation-defective SUMO-GA. The incubation at 30°C resulted in S-La after an hour (upper blot), whereas the incubation at 37°C results in an overall weaker sumoylation of La after 1.5 hours (lower blot). C) The IVSA of the La protein depends on ATP as an energy donor. The in vitro sumoylation assay was performed in the presence and absence of ATP and in the presence of the non-hydrolysable γATP analog. The native La and S-La species are indicated on the left, the molecular weights in kDa on the right. SE = short exposure, IB = immunoblot, S-La = sumoylated La). (TIF) [file pone.0156365.s001.tif]

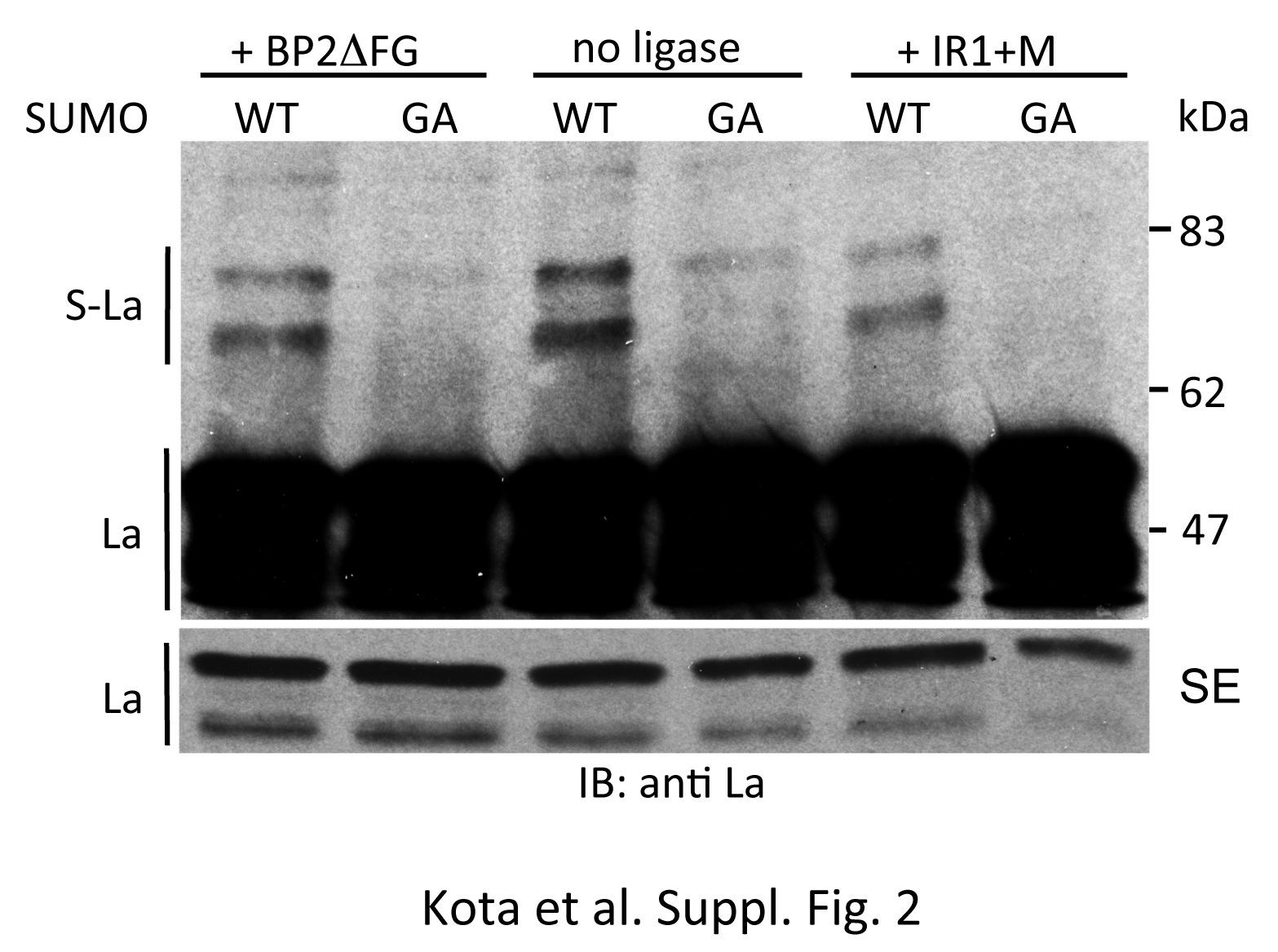

Supplement: S2 Fig — In order to determine if RanBP2 ligase activity can increase the efficiency of the in vitro sumoylation assay of recombinant human La, two minimal functional recombinant RanBP2 proteins (BP2DFG and IR1+M) were purified and used in the IVSA. The sumoylation assay was carried out for 2 hours at 30°C and samples were analyzed as described. Sumoylated La (S-La) is indicated on the left. Modification of La is observed only when SUMO-WT is used and not in the presence of the conjugation-deficient SUMO-GA mutant. The short exposure (SE) shows similar La levels (lower blot). The molecular weight (kDa) is indicated on the right. (TIF) [file pone.0156365.s002.tif]

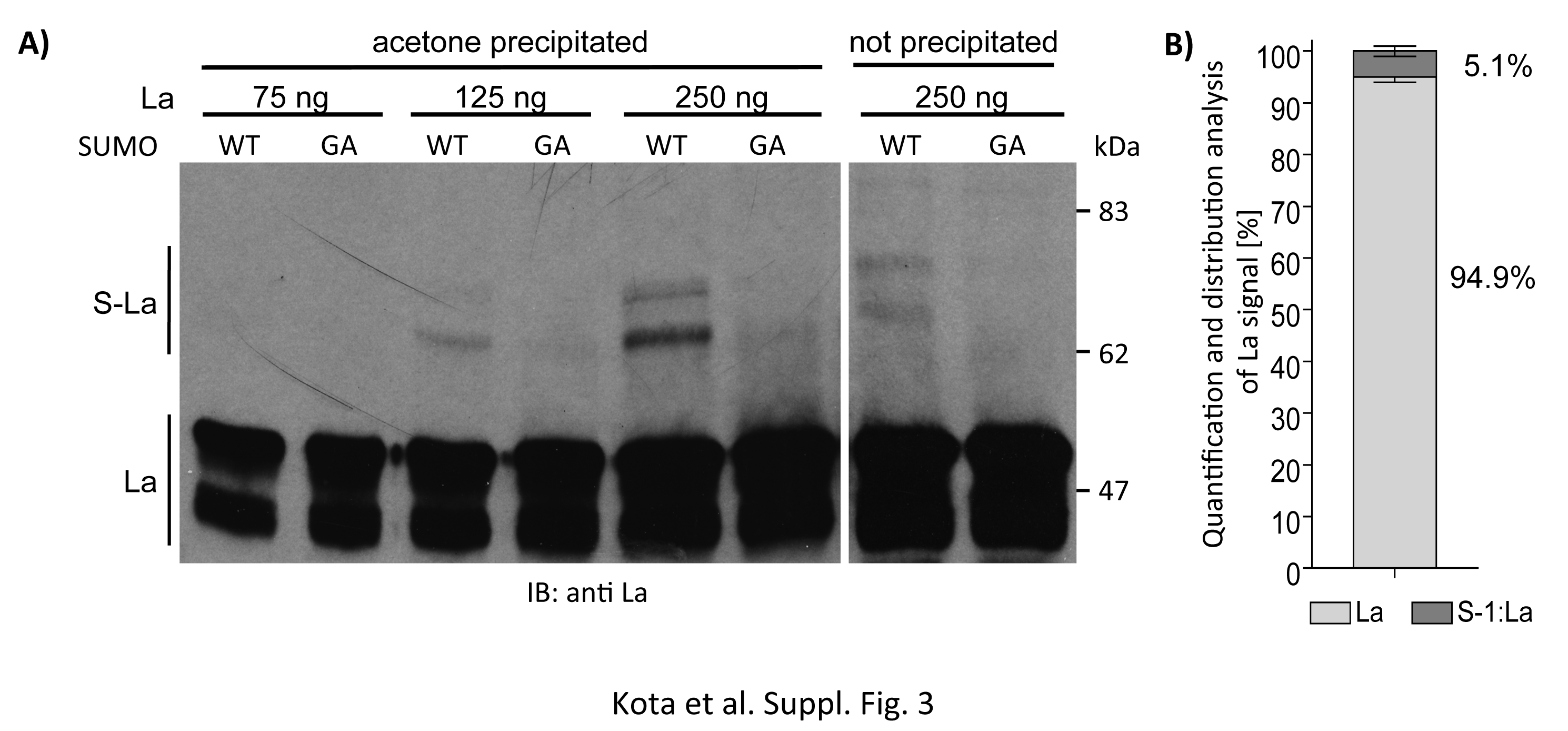

Supplement: S3 Fig — To allow the analysis of the complete in vitro sumoylation assay products and to enhance the immunodetection, samples were precipitated with acetone overnight. A) In vitro sumoylation assay was performed and aliquots of different La amounts (50, 125, and 250 ng) were taken and precipitated overnight followed by immunoblot (IB) analysis for La. Sumoylated La (S-La) is indicated on the left. B) Immunoblot quantification of in vitro sumoylation assay products revealed that on average only 5% of total La is sumoylated. Chemiluminescence signals of four independent in vitro sumoylation assay products were recorded (n = 4) and quantified using the ImageQuant RT ECL instrument and ImageQuant TL software. (TIF) [file pone.0156365.s003.tif]

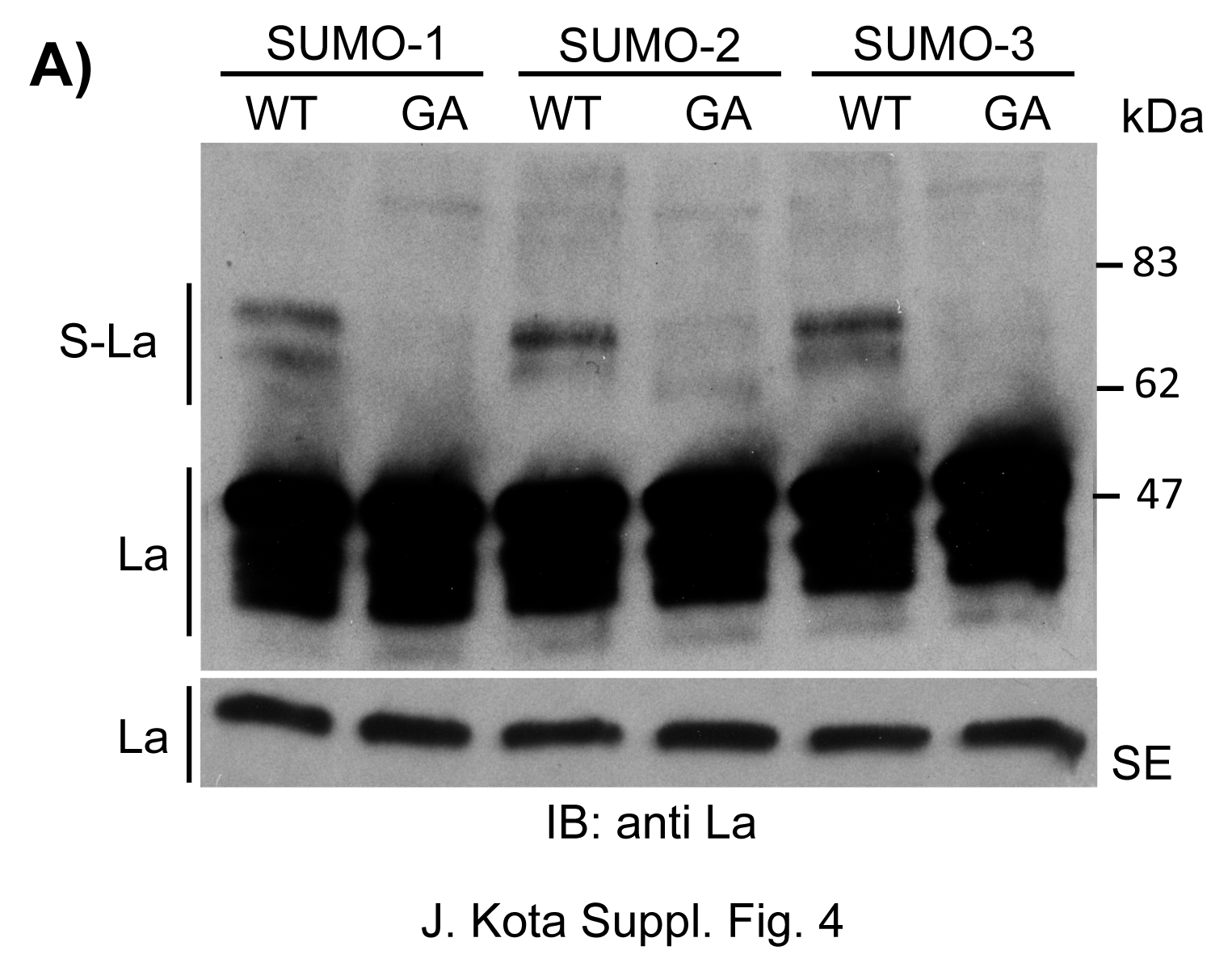

Supplement: S4 Fig — A) Immunoblot (IB) analysis for La shows La-specific bands representing sumoylated La (S-La) in samples with all three SUMO-WT paralogs, but not in conjugation-incompetent SUMO-GA controls. SE = Short Exposure. (TIF) [file pone.0156365.s004.tif]

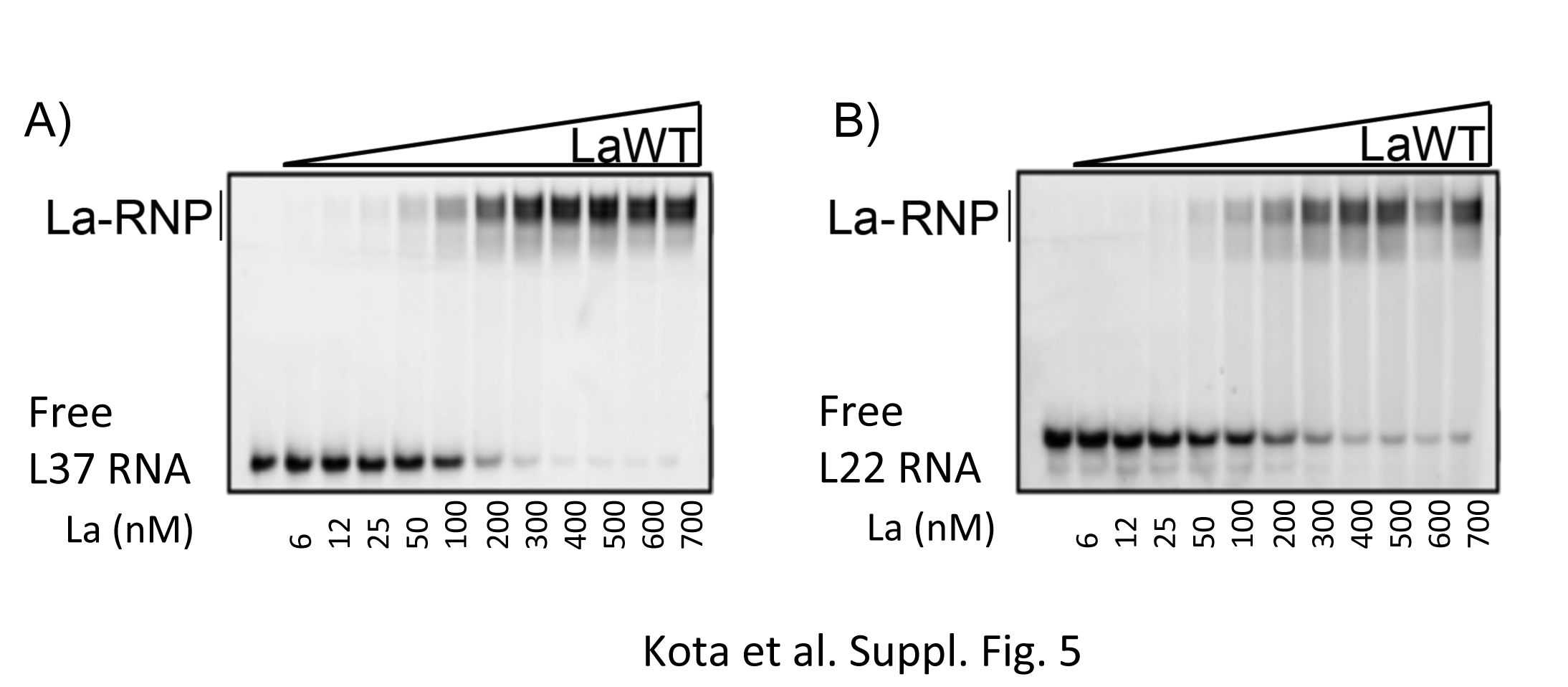

Supplement: S5 Fig — To study the binding of La to L37 and L22 RNA, Cy3-labeled RNA-oligonucleotides representing the 5’-terminal 37 nts of rat L37 and L22 were synthesized. For the binding reactions 22 nM of the RNA oligonucleotides and 6.0, 12.5, 25, 50, 100, 200, 300, 400, 500, 600, and 700 nM of recombinant human La was titrated. Fluorescence of Cy3-labeled free RNA and La-bound RNA were detected using a Typhoon Imager. No La protein was added in the first lane. (TIF) [file pone.0156365.s005.tif]

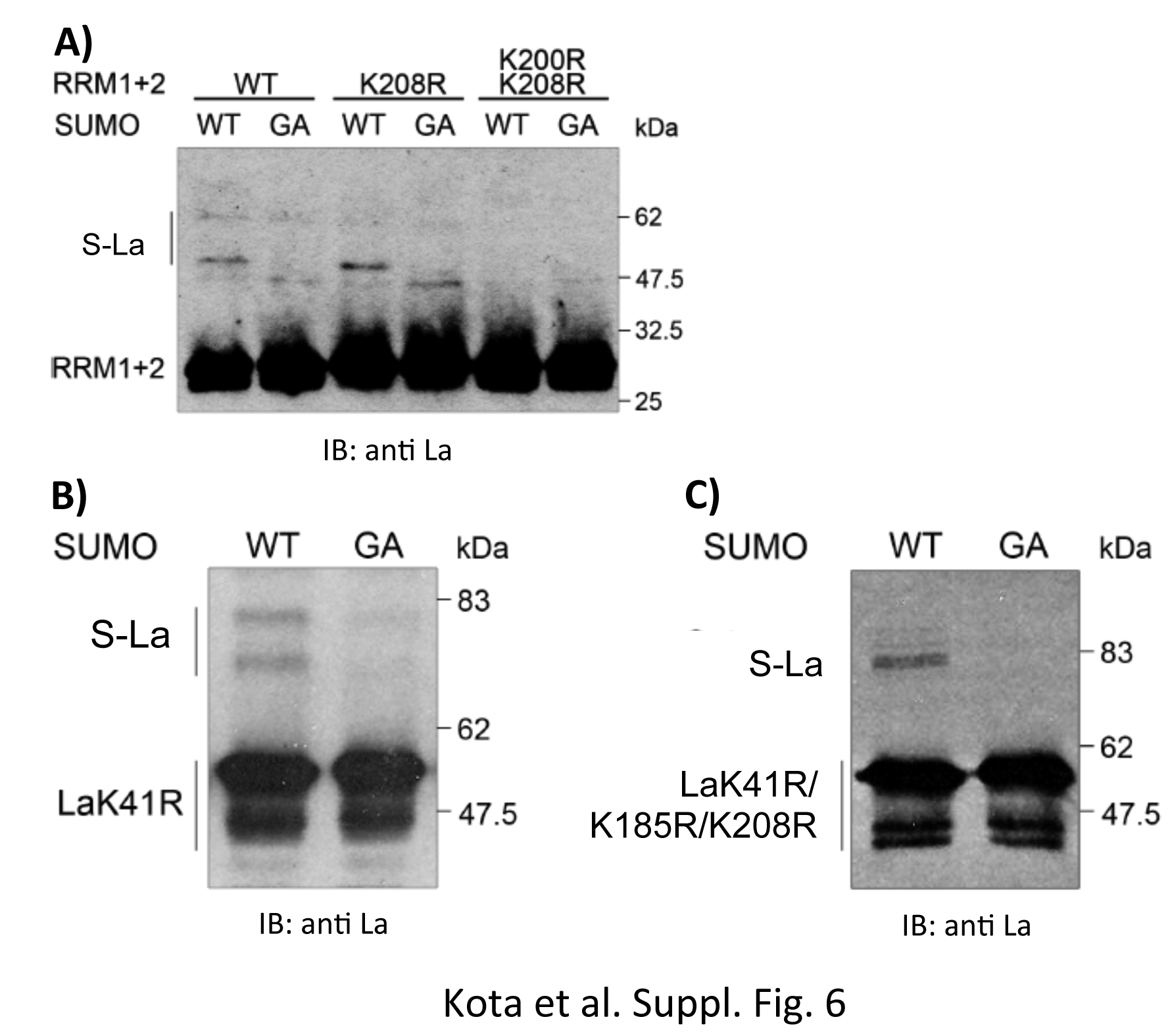

Supplement: S6 Fig — In vitro sumoylation assay was carried out for 2 hours at 30°C, reactions were subjected to immunoblot (IB) analysis for La. The following La mutants with lysine to arginine substitutions were tested: A) RRM1+2K208R and RRM1+2K200R/208R; B) LaK41R; C) LaSM123 (triple mutation of K41R/195R/208R). Immunoblot (IB) analysis shows La-specific bands between 62 and 83 kDa (S-La). Those bands were not detectable with reaction-incompetent SUMO-GA. (TIF) [file pone.0156365.s006.tif]
